# Correction, Vol. 10, No. 6

**DOI:** 10.3201/eid1009.C21009

**Published:** 2004-09

**Keywords:** errata, erratum, correction

Correction heading for “Distribution of Bovine Spongiform Encephalopathy Infectivity in Greater Kudu (Tragelaphus strepsiceros)” by Andrew A. Cunningham et al. was inaccurate. Article appeared in Vol. 10, No. 6. online at http://wwwnc.cdc.gov/eid/article/10/6/03-0615_article.htm.

We regret any confusion these errors may have caused.

